# Perceived organizational politics and organizational performance in governmental organizations: the moderating role of leadership styles

**DOI:** 10.3389/fpsyg.2026.1838756

**Published:** 2026-09-17

**Authors:** Chan Nyein Aung, Xinwen Zhang

**Affiliations:** College of Public Administration, Nanjing Agricultural University, Nanjing, China

**Keywords:** organizational performance, perceived organizational politics, public manager, transactional leadership, transformational leadership

## Abstract

Effective leadership is essential for mitigating the adverse effects of organizational politics and enhancing organizational performance in public organizations. However, empirical evidence on the relationship between perceived organizational politics and organizational performance, as moderated by leadership styles, remains limited. This study examines how the perception of organizational politics influences organizational performance and investigates the moderating role of different leadership styles. Data were collected from 178 public managers of governmental organizations in Myanmar and analyzed using SPSS and structural equation modeling. The results further show that perceived organizational politics is negatively associated with organizational performance. The findings also indicate that leadership styles differ in the extent to which they moderate the relationship between perceived organizational politics and organizational performance. Specifically, transformational leadership significantly moderates this relationship; however, the direction of the interaction is opposite to the hypothesized buffering effect, such that the negative association between perceived organizational politics and organizational performance becomes stronger at higher levels of transformational leadership. In contrast, transactional leadership does not exhibit a significant moderating effect. Comparative analysis further suggests that the moderating effects of transformational and transactional leadership differ significantly. These findings suggest that public organizations should strengthen the development of context-appropriate leadership styles to improve organizational performance and reduce the harmful effects of organizational politics. Finally, the study develops a conceptual framework that links perceived organizational politics, organizational performance, and leadership styles within the context of the public sector.

## 1 Introduction

Perceived organizational politics (POP) has long been recognized as an important factor shaping employee attitudes, behaviors, and organizational outcomes (Vigoda, 2001; Cheong and Kim, 2022). Public organizations engage in POP more frequently than the private sector (Albloush et al., 2021; Bodla and Danish, 2008), resulting in diminished citizen trust in government and reduced effectiveness of public organizations (Vigoda-Gadot, 2007). This phenomenon is often a result of the stagnant, bureaucratic nature of public organizations, which can hinder their ability to adapt and perform effectively (Edler and Yeow, 2015). Existing research shows that POP is associated with a range of adverse consequences, including higher stress, lower job satisfaction, reduced organizational commitment, weaker job involvement, and stronger turnover intentions (Bodla and Danish, 2008; Khan et al., 2021; Shrestha, 2021). At the organizational level, POP has also negatively impacted the organizational performance of local government and public service organizations (Albloush et al., 2025; Cheong and Kim, 2022). Therefore, understanding POP is essential for addressing the barriers to enhancing public organizational performance.

Organizational performance in the public sector is a multidimensional and difficult construct (Kim, 2004). Unlike private organizations, where performance can often be assessed primarily through financial outcomes, public organizations are expected to achieve broader administrative and societal goals. “Organizational performance is a socially constructed phenomenon that is subjective, complex, and particularly hard to measure in the public sector” (Brewer and Selden, 2000). Consistent with this view, public sector organizational performance should be assessed not only through objective outcomes, such as quantity, effectiveness, and delivery speed, but also through subjective evaluations (Boyne, 2004). In this respect, effectiveness, efficiency, and fairness are especially relevant dimensions of governmental organizational performance because they reflect the core normative and operational objectives of public administration (Asencio, 2016; Kim, 2004; Myaing and Lim, 2024). In a government organization, POP was negatively related to organizational performance, particularly with effectiveness, efficiency, and fairness (Cheong and Kim, 2017). Therefore, mitigating POP or alleviating its consequences is crucial for improving organizational performance. In contrast, effective leadership can enhance organizational productivity and governmental performance (Asencio, 2016; Bass and Avolio, 2004). Specifically, transformational and transactional leadership significantly boost the performance of the public sector (Asencio, 2016; Atiku et al., 2023). So, understanding how POP and leadership influence organizational performance is essential for improving the functioning of governmental organizations.

POP can interfere with organizational processes, leading to reduced productivity and performance. The weak organizational performance in public organizations may be attributed to organizational politics and the impact of ineffective leadership (Al-Okaily et al., 2024). Leadership plays an essential role in mitigating the adverse effects of POP. A substantial body of public leadership research suggests that leadership shapes employee perceptions, administrative behavior, and organizational outcomes (Asencio, 2016; Bass and Avolio, 2004). In particular, transformational and transactional leadership have received sustained attention in both general management and public administration scholarship (Backhaus and Vogel, 2022; Nguyen et al., 2022). Transformational leadership, through vision, inspiration, individualized consideration, and intellectual stimulation, may reduce the harmful effects of POP by fostering fairness, transparency, trust, and engagement (Vigoda-Gadot, 2007; Kakhki et al., 2024). Transactional leadership, by emphasizing contingent reward, role clarity, and performance monitoring, may also influence how employees interpret political processes and how such perceptions translate into organizational outcomes. Moreover, prior research has shown that leadership can moderate the perception of organizational politics and organizational outcomes (Saleem, 2015). Studies on servant and authentic leadership likewise indicate that leadership can buffer the negative consequences of perceptions of organizational politics by fostering supportive and ethically grounded organizational environments (Asif et al., 2025; Pasricha, 2025). However, despite these advances, the moderating role of transformational and transactional leadership in the relationship between POP and organizational performance remains insufficiently examined, particularly in public sector settings.

Organizational politics are prevalent in public institutions and can influence their performance (Vigoda, 2001). Such political factors shape organizational objectives, operations, and processes (Farazmand, 1999). However, two important gaps remain in the literature. First, the majority of the existing literature on the relationship between perceptions of organizational politics and organizational performance has been conducted in developed-country settings (Cheong and Kim, 2022; Vigoda-Gadot, 2007). Therefore, further research is still needed to validate how POP impacts organizational performance in the developing countries’ contexts. Second, previous scholars have examined the moderating role of transformational, authentic, and servant leadership on the relationship between POP and various outcomes separately (Kakhki et al., 2024; Pasricha, 2025; Rodriguez et al., 2024; Sarpong et al., 2025). Comparatively little is known about whether different leadership styles, particularly transformational and transactional leadership, differ in the extent to which they mitigate the negative effect of POP on organizational performance in the public sector. Accordingly, this study addresses an important theoretical gap by investigating how different leadership styles moderate the relationship between POP and OP in the public sector context. This analysis contributes to leadership research by comparing the relative moderating roles of transformational and transactional leadership, while also providing evidence relevant to public managers seeking to reduce the harmful consequences of organizational politics and improve organizational performance.

Against this background, the research aims to analyze whether leadership styles can mitigate the negative relationship between perceived organizational politics and organizational performance. The specific objectives of the study are: (i) to examine whether leadership styles moderate the relationship between perceived organizational politics and organizational performance, and (ii) to compare the relative moderating effects of transformational leadership and transactional leadership on the POP and OP relationship. The purpose of this research is to provide empirical evidence on how the perception of organizational politics, along with the moderating influence of leadership, shapes organizational performance in the governmental organization. Based on the findings, it offers empirical evidence on how perceived organizational politics and leadership styles jointly shape public organizational performance and offers practical implications for strengthening effectiveness, efficiency, and fairness in governmental organizations.

## 2 Literature review and hypotheses development

### 2.1 Perceived organizational politics

Perception of organizational politics (POP) is a widely recognized factor in organizational behavior that influences both employee and organizational performance (Asif et al., 2025; Al-Okaily et al., 2024). POP refers to “involves actions by individuals that are directed toward the goal of furthering their own self-interests, without regard for the well-being of others or their organization” (Kacmar and Baron, 1999). Research has consistently shown that the level of POP is higher in public organizations compared to private sector organizations (Vigoda-Gadot, 2007), primarily due to their susceptibility to workplace favoritism, self-serving behavior, and power conflicts. These political dynamics are particularly prevalent in governmental organizations, where strong bureaucratic traditions and seniority-based hierarchies prevail. Empirical research has demonstrated that high levels of POP are associated with negative outcomes, including diminished individual and organizational performance, lower motivation, higher levels of burnout, reduced job satisfaction, increased turnover intention, and lower organizational commitment (Asif et al., 2025; Vigoda-Gadot, 2007). For instance, Al-Okaily et al. (2024) found that both individual and organizational performance are significantly hindered by the negative consequences of POP within the public sector. As demonstrated by previous studies, POP harms employees’ job satisfaction, increases burnout, fosters turnover intention, and commitment, all of which contribute to reduced organizational performance (Vigoda-Gadot, 2007). Therefore, it is evident that organizational performance is negatively related to perceived organizational performance.

The adverse effects of POP are extensively established, and leadership styles are also known to influence how POP is perceived and its impact on organizational outcomes. Transformational leadership helps to mitigate the negative effects of POP (Dappa et al., 2019; Vigoda-Gadot, 2007). The transformational style energizes ethics and promotes optimism by diminishing feelings of dependency (Kacmar and Ferris, 1991). Previous studies have highlighted that transformational leadership can effectively reduce POP by promoting transparency, fairness, and employee engagement, which in turn improves organizational outcomes (Durrani, 2014; Vigoda-Gadot, 2007).

In contrast, transactional leadership demonstrates powerful organizational politics, characterized by its ability to motivate people through various rewards and the application of cooperative methods aligned with the organization’s political environment (Durrani, 2014). Empirical evidence suggests that transactional leadership is associated with both positive and negative effects on POP. Some studies have found that transactional leadership adversely affects POP (Zafar, 2023), while others suggest TSL has a positive impact on POP (Durrani, 2014). This mixed evidence suggests that the impact of transactional leadership on POP is contingent upon the context.

Moreover, previous research has shown that perceived organizational performance is inversely associated with transformational leadership, while the relationship between transactional leadership and POP is both positively and negatively influenced by POP. This complexity indicates that although both leadership styles can influence POP, transformational leadership generally exhibits a more stable and beneficial effect on alleviating the adverse consequences of POP, while transactional leadership may yield more inconsistent outcomes based on the organizational context. The literature shows that while both transformational and transactional leadership styles are related to organizational politics, transformational leadership minimizes the detrimental effects of POP on organizational performance better. However, the impacts of TSL are ambiguous, highlighting the necessity for additional study to clarify its function in moderating the relationship between POP and organizational outcomes. The structure of the proposed research model is presented in Figure 1. The following hypothesis is developed based on the above.

**Figure 1:**
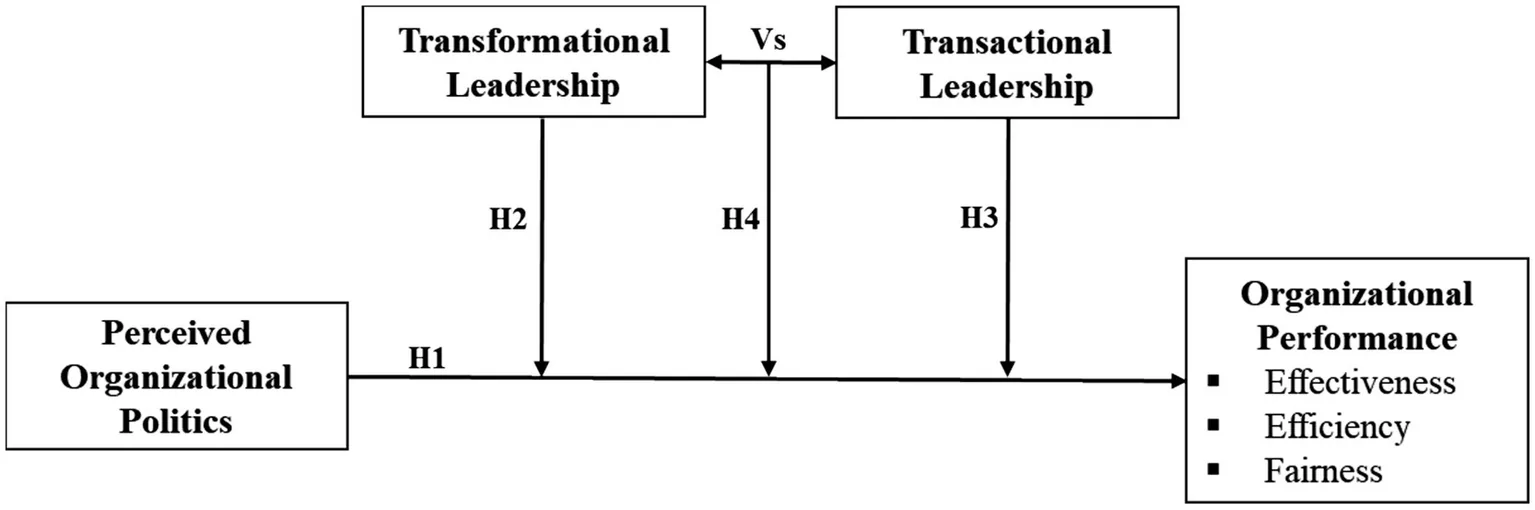
Theoretical framework.

*Hypothesis 1.*. There is a negative relationship between the perception of organizational politics and organizational performance, such that higher levels of POP are associated with lower OP

### 2.2 Organizational performance

Enhancing performance in governmental organizations has emerged as a key focus under the Sustainable Development Goals (SDGs), particularly in achieving peace, justice, and strong institutions. Additionally, performance has been a primary concern in the New Public Management (NPM) framework, which emphasizes effectiveness, efficiency, and fairness within public organizations. These dimensions are crucial for public sectors, as they directly impact the achievement of organizational goals, the optimization of resource use, the preservation of public trust and legitimacy, and assurance of equitable service delivery (Asencio, 2016; Kim, 2004). Empirical studies have demonstrated that organizational performance is influenced by both organizational politics and leadership styles, with each factor contributing to the outcomes of public organizations.

Brewer and Selden (2000) developed a framework for governmental performance measurement composed of the internal and external factors on three administrative values: efficiency, effectiveness, and fairness (Brewer and Selden, 2000). This framework has been successfully employed by multiple scholars, particularly to evaluate organizational performance in governmental organizations (Kim, 2022), organizational performance (Asencio, 2016), and public service performance (Myaing and Lim, 2024). The effectiveness, efficiency, and fairness serve as a vital evaluation tool for assessing performance related to public organizations. The effectiveness of public organizations is measured by the successful implementation of public policies and achievement of organizational goals (Andrews et al., 2017). Efficiency refers to the effectiveness with which an organization utilizes its financial, human, and material resources to attain its goals (Andrews et al., 2017). Fairness is defined as an equitable allocation of resources, benefits, and public services to all individuals, particularly marginalized groups in society (Andrews et al., 2017). These dimensions collectively offer a comprehensive framework for evaluating public organizations, providing insights into program operations, management practices, and service delivery outcomes.

Given the challenges of obtaining objective data in some contexts, particularly within governmental organizations, subjective performance measures are often employed as an alternative to objective data. While objective measures are less biased, they are difficult to obtain due to the constraints of public sector settings. Public employees’ perceptions of organization performance are valid, reliable, and sensitive (Andrews et al., 2017). The subjective measures are frequently used as a substitute for objective data (Brewer and Selden, 2000). Recent studies have highlighted the utility of subjective measures in assessing public sector performance, reflecting the views of employees on the overall effectiveness of their organizations (Asencio, 2016; Cheong and Kim, 2022; Kim, 2022). Therefore, this study employed the subjective measurement of organizational performance, which reflects the perceptions of public employees regarding the overall performance of their organizations.

### 2.3 Moderating effect of transformational and transactional leadership

Leadership styles play an important moderating role in the relationship between perceived organizational politics and organizational performance. The Full Range of Leadership Model (FRLM) provides a prominent theoretical framework for explaining the organizational consequences of different leadership styles, particularly in public sector settings (Bass and Avolio, 2004; Trottier et al., 2008). Within this framework, transformational and transactional leadership have received considerable attention in public administration research (Backhaus and Vogel, 2022; Nguyen et al., 2022). Existing studies have more frequently employed transformational leadership as a moderating variable to examine organizational climate and adverse interpersonal outcomes (Islam et al., 2021). By contrast, relatively limited research has incorporated transactional leadership as a moderator in organizational behavior models (Ha and Moon, 2023). In addition, effective leadership styles may reduce employees’ perceptions of organizational politics and mitigate their harmful consequences (Saleem, 2015). Accordingly, this study draws on the FRLM to examine how transformational and transactional leadership shapes the relationship between perceived organizational politics and organizational performance.

First, transformational leadership is characterized by leaders who inspire, motivate, innovate, and encourage subordinates to achieve outstanding results, foster positive change, and fulfill anticipated outcomes (Ofosu-Anim, 2022). TFL is operationalized through five essential dimensions: idealized influence (attributes), idealized influence (behaviors), inspirational motivation, intellectual stimulation, and individualized consideration (Bass and Avolio, 2004). The main aim of the TFL is to inspire and motivate followers to perform at their highest capacity and meet expectations. This leadership style is applied when an organization necessitates revitalization, is undergoing substantial change, or seeks a new direction (Benmira and Agboola, 2021). Extensive literature has demonstrated that transformational leadership is substantially correlated with both individual-level outcomes (such as motivations, attitudes, and behaviors) and organizational-level outcomes (such as performance and innovation) (Bao et al., 2024). Prior empirical research has shown that transformational leadership is positively and significantly correlated with organizational performance (Asencio, 2016; Chau et al., 2022; Makatita et al., 2024). Additionally, transformational leadership has a negative influence on perceived organizational politics (Vigoda-Gadot, 2007; Durrani, 2014), thereby enhancing organizational performance.

Prior research also suggests that transformational leadership can buffer the negative effects of perceived organizational politics. For example, transformational leaders can moderate negative perceptions of organizational politics by fostering a positive perspective on knowledge management development, collaborating to address professional challenges, motivating the proper execution of knowledge management responsibilities, and facilitating cooperation in knowledge management decision-making (Kakhki et al., 2024). Similarly, the probability that higher levels of POP reduce OCB was consequently diminished when employees could rely on transformative leaders (De Clercq and Belausteguigoitia, 2017). Vigoda-Gadot and Beeri (2011) observed that transformational leadership mitigated the adverse effects of POP on job satisfaction. Furthermore, recent studies have indicated that TFL also moderates the negative effect of perception of organizational politics on physical, social, and environmental well-being (Sarpong et al., 2025). Prior research also indicated that trust in fellow workers and social support moderate the relationship between POPs and job outcomes within a university context (Vigoda-Gadot and Talmud, 2010). These findings suggest that transformational leadership is a crucial moderator of the relationship between POP and OP. Therefore, TFL may significantly moderate the relationship between POP and OP, especially by creating a more supportive, transparent, and motivating organizational environment. Thus, the following hypothesis is proposed.

*Hypothesis 2*. The negative relationship between POP and OP is moderated by TFL, such that the relationship weakens as transformational leadership increases.

Second, transactional leadership involves an exchange between leaders and subordinates, wherein leaders acknowledge subordinates’ demands and offer incentives and recognition to motivate them (Asencio, 2016; Bass, 1996). Unlike transformational leadership, which emphasizes vision, intrinsic motivation, and value alignment, transactional leadership focuses on establishing clear performance standards, maintaining organizational order, and ensuring that desired outcomes are achieved through structured exchanges (Aarons, 2006; Benmira and Agboola, 2021). TSL is typically divided into two dimensions: contingent reward management by exception (active) (Serrat, 2021). Bass and Avolio (2004) suggest that contingent rewards offer a reward for effort and recognize good performance through random rewards (contingent positive reinforcement) or contingent punishment (contingent negative reinforcement). This dimension emphasizes clearly defined roles, procedures, and performance standards, such that employees who meet or exceed expectations receive appropriate rewards. In management-by-exception, employees are monitored and supervised to ensure adherence to work procedures, hence facilitating the completion of leadership tasks (Thanh and Van Quang, 2022). More broadly, transactional leadership emphasizes goal attainment, role clarification, and performance monitoring; it is often regarded as a practical leadership approach (Aarons, 2006). TSL is particularly effective in organizations that are mature, with clearly defined structures and goals to keep them on track and preserve their status quo (Benmira and Agboola, 2021). However, its effectiveness depends greatly on the leader’s ability to respond appropriately: poor transactional leaders may struggle to anticipate issues and intervene preemptively, whereas effective transactional leaders act decisively and promptly (Aarons, 2006). At the same time, research suggests that transactional leadership exhibits both beneficial and detrimental indirect impacts on task and contextual performance (Young et al., 2020), indicating that its influence may vary across organizational conditions and employee outcomes.

Perceived organizational politics reflects employees’ perceptions that organizational decisions, resource allocation, promotions, or rewards are influenced by self-serving behaviors rather than objective criteria. Such perceptions often generate uncertainty, reduce trust, and negatively affect employee attitudes and organizational outcomes. From this perspective, transactional leadership may influence the POP–OP relationship by providing employees with clearer performance criteria, explicit reward structures, and predictable evaluation processes. By strengthening the connection between effort, performance, and outcomes, transactional leaders may reduce ambiguity regarding how employees are assessed and rewarded, thereby potentially mitigating some negative consequences associated with perceived organizational politics.

Empirical evidence indicates that transactional leadership is associated with a range of organizational outcomes, although its relationship with perceived organizational politics remains theoretically complex. Transactional leadership can enhance organizational performance through goal clarification and performance monitoring (Asencio, 2016; Makatita et al., 2024) excessive reliance on control mechanisms, formal exchanges, or reward-based relationships may also reinforce employees’ sensitivity to political behaviors, particularly when rewards and evaluations are perceived as unfair. Additional research indicates that TSL is positively associated with perceptions of improved work quality, cooperation, and job satisfaction (Oberfield, 2012), as well as fulfillment of employee needs and increased organizational performance (Nguyen et al., 2022). Accordingly, prior research has reported mixed findings regarding the relationship between transactional leadership and perceived organizational politics. For example, Zafar (2023) found that transactional leadership had a significant negative impact on perceived organizational politics in the private sector of Pakistan. Conversely, Saleem (2015) reported a significant positive relationship between transactional leadership and perceived organizational politics in public sector universities of Pakistan. These inconsistent findings suggest that the influence of transactional leadership on political perceptions may depend on organizational context and may not be uniformly beneficial.

As a moderating variable, transactional leadership appears to have a more ambiguous role than transformational leadership. Prior studies suggest that transactional leadership can moderate work-related relationships, although the direction and strength of this effect vary. For instance, prior studies indicate that an increased perception of the leader as transactional correlates with lower positive relationships between perceived organizational politics and the interpersonal aspect of organizational citizenship behaviors (Tziner et al., 2021). Further study reveals that transactional leadership mitigates the negative correlation between distributive justice and organizational citizenship behaviors (Ha and Moon, 2023). The increased level of TSL enhanced the positive impact of job description on employee performance within the public sector (Rawas and Jantan, 2023). However, no previous study has examined how transactional leadership moderates the relationship between POP and OP within public organizations. Therefore, building on the FRLM and the existing empirical evidence, this study proposes that transactional leadership may weaken the negative effect of perceived organizational politics on organizational performance by promoting role clarity, monitoring performance, and reinforcing goal-oriented behavior. Therefore, the following hypothesis is proposed.

*Hypothesis 3*. The negative relationship between POP and OP is moderated by TSL, such that the relationship weakens as transactional leadership increases.

### 2.4 Comparing the moderating effect of different leadership styles

Empirical research indicates that leadership styles may moderate organizational relationships in different ways and with different magnitudes. For instance, Ha and Moon (2023) found that transactional leadership strengthened the positive relationship between distributive justice and organizational citizenship behavior (OCB), whereas transformational leadership strengthened the positive relationship between goal clarity and OCB. Their findings suggest that the magnitude and direction of leadership effects depend on both the leadership style and the underlying organizational mechanism. This provides a crucial foundation for understanding that transformational and transactional leadership may vary in their capacity to mitigate the adverse relationship between perceived organizational politics and organizational performance. Empirical research further suggests that transformational and transactional leadership may not exert equivalent effects when organizational politics is taken into account. For instance, Budiasih et al. (2020) reported that POP mediated the relationship between transformational leadership and job satisfaction as well as the relationship between transactional leadership and job satisfaction, although the magnitude of these effects differed. Similarly, Saleem (2015) showed that when POP was included in the model, the effect of transformational leadership on job satisfaction declined from *β* = 0.638 to *β* = 0.522 (a change of nearly 0.116), whereas the effect of transactional leadership changed from *β* = −0.285 to *β* = −0.135 (a change of nearly 0.150). These findings indicate that perceived organizational politics alters the strength of the relationships between leadership styles and work outcomes, and that this influence is not identical across leadership styles. Although these studies focused on mediation rather than moderation, they nevertheless suggest that transformational and transactional leadership function differently when organizational politics is involved.

The existing literature suggests that different leadership styles may reduce the adverse effects of organizational politics to different degrees. Despite this theoretical expectation, limited research has directly compared the moderating effects of transformational and transactional leadership on the relationship between POP and organizational performance, particularly in public organizations. Therefore, the present study proposes the following hypothesis.

*Hypothesis 4*. The moderating effects of transformational and transactional leadership on the negative relationship between perceived organizational politics and organizational performance differ significantly.

## 3 Materials and methods

### 3.1 Sampling and procedures

The research utilized a quantitative survey design to test the proposed hypotheses. Data were collected from public servants working in governmental organizations in Myanmar between April and June 2025. The survey focused on general-service public managers (PM) from the General Administration Department (GAD) across Myanmar’s 15 states and regions. Public managers in the GAD represent a key administrative authority, as they serve as frontline actors in policy implementation and public service delivery throughout the country (Saw and Arnold, 2014). According to Saw and Arnold (2014), approximately 330 public managers were appointed within the GAD nationwide. Based on this population, a total of 330 questionnaires were purposively distributed in Myanmar. Of the 330 questionnaires distributed, 185 were returned, resulting in a response rate of 56%. After data screening and cleaning, seven responses were excluded due to substantial missing data; 178 questionnaires were retained for analysis, representing 95% of the returned surveys. The respondents represented different administrative levels, including state and region capitals (11.2%), districts (26.4%), and townships (62.4%). Because purposive rather than probability sampling was employed, the sample should not be interpreted as statistically representative of the entire Myanmar public sector; rather, it provides substantial coverage of the specific population of GAD public managers examined in this study. Before administering the survey, we informed the participants of the purpose of the research and their right to participate voluntarily or withdraw at any time without penalty. To mitigate the likelihood of nonresponse bias and social desirability bias, participants were guaranteed that their responses would be treated confidentially, their anonymity would be protected, and no pressure would be placed on them to provide favorable responses. Informed consent was obtained before the distribution of the questionnaires. Consent was secured through both oral communication and written online correspondence. The processes aimed to guarantee that participants comprehended the study’s nature and their rights as respondents prior to completing the questionnaire.

### 3.2 Measure instruments

The survey instrument included 51 items organized into four sections: demographic profile, perceived organizational politics, organizational performance, and leadership styles (Appendix A). For the independent variable, the research employed the perception of organizational politics scale developed by Vigoda (2001) to assess perceived organizational politics. POP refers to “the degree to which the respondents view their work environment as political, and therefore unjust and unfair”. The instrument consisted of six items, and the reliability for the POP scale was acceptable (Cronbach’s α = 0.79). POP was measured on a five-point Likert scale ranging from 1 (“strongly disagree”) to 5 (“strongly agree”). Samples included items such as “Favoritism rather than merit determines who gets ahead around here” and “There is a group of people in my department who always get things their way because no one wants to challenge them”.

For the dependent variable, the study utilized the governmental performance instrument developed by Brewer and Selden (2000) and Kim (2004) to measure organizational performance. OP is defined as “the internal and external effectiveness, efficiency, and fairness of an organization”. The instrument included three factors with twelve survey questions: effectiveness, efficiency, and fairness. Each factor comprises four items. The reliability of the OP scale was strong (Cronbach’s α = 0.87). OP was measured on a five-point Likert scale ranging from 1 (“strongly disagree”) to 5 (“strongly agree”). For example, “My organization has made good use of my knowledge and skills in looking for ways to become more efficient” and “My organization provides fair and equitable services to the public without considering their backgrounds”.

For the moderating variables, the study employed the self-report leader form of MLQ-5X developed by Bass and Avolio (2004) to measure the transformational and transactional leadership styles (Appendix B). Accordingly, the leadership measures represent public managers’ evaluations of their own leadership behaviors rather than subordinate ratings of their leaders. TFL refers to “building trust, acting with integrity, encouraging others, encouraging innovative thinking, and coaching and developing people” (Serrat, 2021). TSL refers to reward achievement and monitors deviations & mistakes (Serrat, 2021). The instrument consists of 28 items: transactional leadership (8 items) and transformational leadership (20 items) (Bass and Avolio, 2004; Serrat, 2021; Barnes et al., 2013). This research employed self-reporting measures (leader form) to provide a specific leader perspective. The internal consistency of the leadership scales was satisfactory for TFL (Cronbach’s α = 0.75–0.84) and TSL (Cronbach’s α = 0.71–0.76) (Bass and Avolio, 2004). Leadership styles were measured using a five-point Likert scale ranging from 0 (“not at all”) to 4 (“frequently, if not always”). Samples involved items such as “I go beyond self-interest” and “I discuss specific performance”.

### 3.3 Data analysis

The data were analyzed using SPSS 25 for descriptive statistics and SmartPLS 4 for partial least squares structural equation modeling (PLS-SEM) (Hair et al., 2018). First, descriptive statistics were performed to summarize the study variables, providing an overview of participants’ responses. Second, the hypothesis testing procedure begins with the evaluation of the measurement model through outer loadings, reliability, and validity assessments. Next, the structural model was analyzed to assess the impact of perceived organizational politics on organizational performance, considering the moderating effect of transformational and transactional leadership. The PLS-SEM approach enables the simultaneous estimation of complex relationships among constructs, including moderation effects, and is particularly suitable for exploratory research and relatively small sample sizes (Hair et al., 2018).

The adequacy of the sample size for PLS-SEM was also considered. Because the commonly cited 10-times rule provides only a rough indication of minimum sample size, we additionally applied the inverse square root method proposed by Kock and Hadaya (2016). This method is recommended for applied PLS-SEM research because of its simplicity and relatively conservative estimation properties. When the minimum expected path coefficient is not known *a priori*, Kock and Hadaya suggest a benchmark of approximately (β = 0.197), corresponding to a minimum required sample size of approximately 160 observations at a 5% significance level and about 80% statistical power. The final sample of 178 respondents exceeded this threshold and was therefore considered adequate for the PLS-SEM analysis. The study also assessed common method variance (CMV) using Harman’s single factor test (Podsakoff et al., 2003). Before conducting the test, the Kaiser–Meyer–Olkin (KMO) measure of sampling adequacy was 0.936, exceeding the recommended threshold of 0.50, and Bartlett’s test of sphericity was significant (χ^2^ = 4032.667, df = 1035, *p* < 0.001), indicating that the data were suitable for factor analysis (Kaiser, 1974; Bartlett, 1954). The unrotated exploratory factor analysis including all substantive measurement items showed that the first factor accounted for 35.92% of the total variance, which was below the commonly referenced 50% threshold (Yu and Wu, 2024). Although we acknowledge that Harman’s single-factor test cannot completely eliminate the possibility of common method variance, the results suggest that CMV is unlikely to be a major concern in this study.

## 4 Results

### 4.1 Descriptive analysis

The demographic profile of the participants, including age, education level, work experience, and duty station, is presented in Table 1.

**Table 1:** Demographic profile of the participants (*N* = 178).

Variables	Content	Quantity	Percentage
Age	25–34 years	13	7.3
	35–44 years	83	46.6
	45–54 years	60	33.7
	55–62 years	22	12.4
Education level	Bachelor	138	77.5
	Master	39	21.9
	Doctoral	1	0.6
Work experience	11–15 years	59	33.1
	16–20 years	38	21.3
	21–25 years	46	25.8
	26–30 years	19	10.7
	Over 31 years	16	9.0
Duty station	State and Region Capital	20	11.2
	District	47	26.4
	Township	111	62.4

The descriptive statistics of the study variables are presented in Table 2. The mediating variables show that transactional leadership has the highest mean score (M = 2.81) compared to transformational leadership (M = 2.74). The dependent variable demonstrated a mean score of organizational performance demonstrated a mean score (M = 3.49), which is slightly above average. The independent variable, perceived organizational politics, reported a mean score (M = 3.11), indicating a slightly above average perception of politics within the organization. The correlations between the variables show that POP has a strong negative correlation with OP (r = −0.762). When the perception of organizational politics increases, organizational performance tends to decrease. Similarly, POP is negatively correlated with TFL (r = −0.798) and TSL (r = −631), indicating that higher levels of organizational politics are associated with lower perceptions of transformational and transactional leadership. Additionally, TFL and TSL positively correlated with OP (r = 0.776, r = 0.667), suggesting that higher levels of transformational and transactional leadership are linked to improved organizational performance.

**Table 2:** Descriptive statistics.

Variable	Items	Mean	SD	Correlations			
				1	2	3	4
1. POP	6	3.11	0.55	1			
2. OP	12	3.49	0.46	−0.762	1		
3. TFL	20	2.74	0.46	−0.798	0.776	1	
4. TSL	8	2.81	0.52	−0.631	0.667	0.681	1

### 4.2 Leadership styles moderate the relationship between POP and OP

PLS-SEM was utilized to investigate whether leadership styles influence the relationship between POP and OP, as it facilitates the analysis of complex relationships among latent variables, including interaction effects, and is suitable for studies with relatively small to medium sample sizes and non-normal data (Goodhue et al., 2012). Before testing the structural model, the measurement model was assessed by examining indicator reliability, internal consistency reliability, convergent validity, discriminant validity, and collinearity. Indicator reliability was evaluated through outer loadings and variance inflation factor (VIF) values Hair et al., 2018. Following PLS-SEM guidelines, indicator loadings above 0.70 are preferred; however, indicators with loadings between 0.40 and 0.70 may be retained when their removal does not improve composite reliability and average variance extracted (AVE), and when they are theoretically meaningful (Hair et al., 2021). In the present study, outer loadings ranged from 0.463 to 0.857, and indicators with extremely low contributions were removed where necessary to improve construct reliability and validity. The retained indicators were theoretically relevant and contributed to satisfactory construct-level reliability. Collinearity was assessed using VIF values. The VIF values ranged from 1.277 to 2.463, which are below the recommended threshold of 3.33 (Hair et al., 2021), indicating that multicollinearity was not a concern. Internal consistency reliability was evaluated using Cronbach’s alpha (CA) and composite reliability (CR). The CA values ranged from 0.84 to 0.92, while CR values ranged from 0.86 to 0.93, exceeding the recommended threshold of 0.70 (Hair et al., 2021). Convergent validity was further supported by average variance extracted (AVE) values ranging from 0.40 to 0.57. Although some AVE values were below the conventional threshold of 0.5, values above 0.4 can still be considered acceptable when the composite reliability (CR) exceeds 0.6 (Fornell and Larcker, 1981). Discriminant validity was assessed using the heterotrait–monotrait ratio (HTMT). Following Henseler et al. (2014), HTMT values below 0.90 indicate adequate discriminant validity. As shown in Table 4, all HTMT values were below the recommended threshold of 0.90, confirming that the constructs represent empirically distinct concepts. The results of the measurement model assessment, including indicator loadings, VIF, reliability, convergent validity, and discriminant validity, are presented in Tables 3, 4 (for more details, see Figure 2).

**Table 3:** Measurement model assessment.

Variables	Indicators	Outer loadings	VIF	CA	CR	AVE
POP	POP1	0.751	1.703	0.84	0.86	0.57
	POP2	0.463	1.277			
	POP3	0.783	1.995			
	POP4	0.801	2.143			
	POP5	0.857	2.463			
	POP6	0.810	2.068			
OP	EEF1	0.664	1.695	0.86	0.86	0.40
	EEF2	0.534	1.332			
	EEFF1	0.618	1.655			
	EEFF2	0.740	2.105			
	EF1	0.622	1.477			
	EF2	0.603	1.428			
	IEF1	0.553	1.446			
	IEF2	0.526	1.330			
	IEFF1	0.650	1.543			
	IEFF2	0.609	1.582			
	IF1	0.667	1.665			
	IF2	0.663	1.715			
TFL	IC2	0.619	1.947	0.92	0.93	0.44
	IC3	0.630	1.626			
	IC4	0.707	2.020			
	IIA2	0.669	2.251			
	IIA3	0.637	1.825			
	IIA4	0.738	2.211			
	IIB1	0.605	1.653			
	IIB2	0.674	1.824			
	IIB3	0.516	1.570			
	IIB4	0.542	1.516			
	IM1	0.742	2.042			
	IM2	0.745	2.119			
	IM3	0.713	2.042			
	IM4	0.557	1.658			
	IS1	0.567	1.547			
	IS2	0.702	1.933			
	IS3	0.734	2.099			
	IS4	0.729	2.355			
TSL	CR1	0.638	1.434	0.85	0.86	0.49
	CR2	0.747	1.761			
	CR3	0.742	1.758			
	CR4	0.718	1.653			
	MBEA1	0.653	1.631			
	MBEA2	0.657	1.537			
	MBEA3	0.718	1.713			
	MBEA4	0.713	1.662			

**Table 4:** Discriminant validity.

Variables	Heterotrait - Monotrait Ratio (HTMT)					
	OP	POP	TFL	TSL	TSL x POP	TFL x POP
OP						
POP	0.894					
TFL	0.866	0.898				
TSL	0.772	0.748	0.758			
TSL x POP	0.215	0.300	0.200	0.138		
TFL x POP	0.410	0.461	0.345	0.186	0.780	

**Figure 2:**
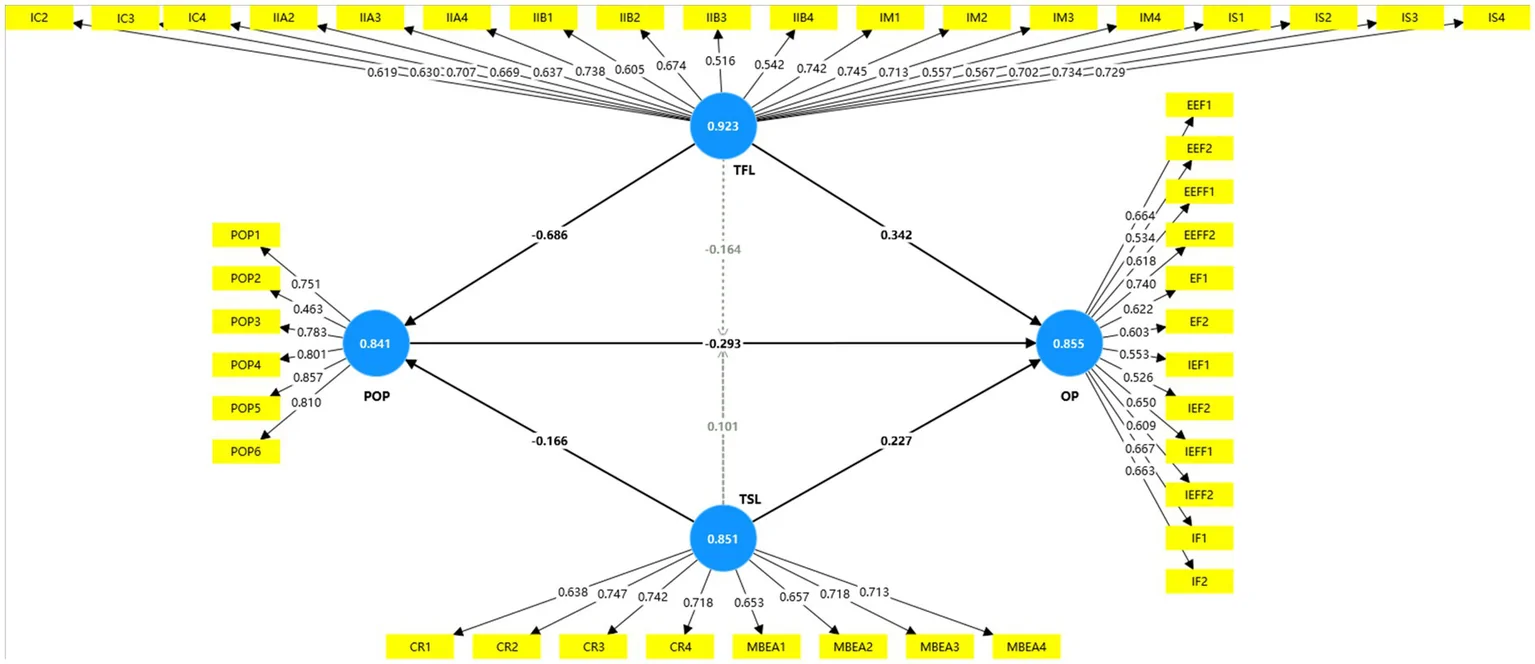
Measurement model assessment.

The findings presented in Table 5 show that the proposed model demonstrates satisfactory explanatory power (R^2^), predictive relevance (Q^2^), and model fit (Chin, 1998). The Q^2^ value of 0.256; the values are greater than zero, indicating that the model has acceptable predictive relevance, with a moderate level of predictive power (Wetzels et al., 2009). A model fit can also be considered acceptable if the standardized root mean square residual (SRMR) value is less than 0.08 (Hu and Bentler, 1999). The proposed model of SRMR yields a value of 0.059, suggesting a good fit between the model and the data. Therefore, the model demonstrates a good fit, adequate predictive relevance, and explanatory power.

**Table 5:** Model good fit result.

Fit Index	OP	POP	Interpretation
R^2^	0.691	0.654	Strong
Adjusted R^2^	0.682	0.650	Strong
Q^2^ predict	0.256	0.364	Moderate
NFI	0.721		Acceptable fit
SRMR	0.059		Good fit

The results of the hypothesis test are shown in Table 6. First, the direct effect results show that perceived organizational politics is significantly related to organizational performance. Specifically, POP has a significant negative impact on OP (*β* = −0.293, *p* = 0.000). It may be plausible that public managers who perceive a higher level of organizational politics experience greater workplace favoritism, power conflicts, uncertainty, distrust, and self-protective behavior, which may undermine organizational performance in terms of effectiveness, efficiency, and fairness, compared to those who perceive a lower level of organizational politics. Therefore, the study supported Hypothesis 1.

**Table 6:** Hypothesis test result.

Hypothesis	Relationship	SB	SD	t value	*p* value	Decision
H1	POP → OP	−0.293	0.072	4.079	0.000	Supported
H2	TFL x POP → OP	−0.164	0.073	2.246	0.025	Not Supported
H3	TSL x POP → OP	0.101	0.074	1.365	0.172	Not Supported

Second, the moderating effect results report that leadership styles differ in the extent to which they shape the relationship between POP and OP. Specifically, transformational leadership significantly moderated the relationship between POP and OP (*β* = −0.164, *p* = 0.025). However, the direction of the interaction was opposite to that predicted in Hypothesis 2. Because the direct POP–OP relationship was negative and the POP × TFL interaction coefficient was also negative, higher levels of transformational leadership were associated with a stronger, rather than weaker, negative relationship between POP and organizational performance. Accordingly, although a statistically significant moderation effect was observed, Hypothesis 2, which specifically predicted that transformational leadership would weaken the negative POP–OP relationship, was not supported.

By contrast, the moderating role of transactional leadership in the relationship between POP and OP is positive but statistically non-significant (*β* = 0.101, *p* = 0.172). The results indicate that transactional leadership does not significantly weaken the negative effect of POP on OP, and thus Hypothesis 3 is not supported. One explanation for this result is that transactional leadership, which emphasizes contingent rewards, performance monitoring, and compliance with established procedures, may be less capable of addressing the uncertainty, distrust, and relational strain associated with perceived organizational politics. Therefore, it does not exert a significant moderating effect on the relationship between perceived organizational politics and organizational performance.

### 4.3 Comparison of the relative magnitude of the moderating effects of transformational and transactional leadership

Table 7 illustrates the comparative moderating effects of transformational and transactional leadership on the relationship between POP and OP. A formal comparison of the two interaction coefficients was conducted using a paired bootstrap procedure. The interaction effect of transformational leadership was negative (*β* = −0.164, *p* = 0.025), whereas the interaction effect of transactional leadership was positive (*β* = 0.101, *p* = 0.172). The difference between the two interaction coefficients was statistically significant (Δβ = −0.265, 95% bootstrap CI [−0.430, −0.081]), as the confidence interval did not include zero. These findings demonstrate that transformational and transactional leadership exert significantly different moderating effects on the relationship between perceived organizational politics and organizational performance. Specifically, transformational leadership produced a more negative moderating effect, whereas transactional leadership showed a comparatively positive moderating effect. Therefore, Hypothesis 4 was supported.

**Table 7:** Comparison of the moderating effects of TFL and TSL.

Hypothesis	Comparison of moderating effects	f^2^ (TFL)	f^2^ (TSL)	β (TFL)	β (TSL)	Δβ	95% Bootstrap CI for Δβ	Decision
H4	POP × TFL → OP vs. POP × TSL → OP	0.041	0.017	−0.164	0.101	−0.265	[−0.430, −0.081]	Supported

The interaction effect sizes were relatively small. The POP × TFL interaction produced f^2^ = 0.041, indicating a small incremental effect, whereas the POP × TSL interaction produced f^2^ = 0.017, indicating a negligible to very small effect (Cohen, 1992). Thus, although the transformational-leadership interaction reached statistical significance, its contribution to explaining additional variance in organizational performance was modest. Statistical significance should therefore not be interpreted as evidence of a large practical effect.

Figures 3A,B present the nature of these interaction effects through simple slope analysis. The two figures demonstrate that the moderating patterns differ across leadership styles. Figure 3A shows that the negative relationship between POP and OP becomes progressively stronger as transformational leadership increases. At low TFL (−1 SD), the POP–OP relationship is comparatively flatter; at the mean level of TFL, the negative slope becomes stronger; and at high TFL (+1 SD), the negative slope is steepest. This pattern is consistent with the negative POP × TFL interaction coefficient (β = −0.164, *p* = 0.025). Thus, transformational leadership significantly moderates the relationship, but in the opposite direction to the buffering effect predicted in Hypothesis 2. In contrast, Figure 3B shows that POP is negatively related to OP across three levels of transactional leadership. At low levels of TSL (−1 SD), the slope is steepest, indicating a strong negative relationship between POP and OP. At the average level, the negative effect becomes moderate. At high levels of TSL (+1 SD), the slope is flatter, indicating a weaker negative relationship. Overall, the results suggest that transformational leadership is more effective than transactional leadership in buffering the negative effect of perceived organizational politics on organizational performance.

**Figure 3:**
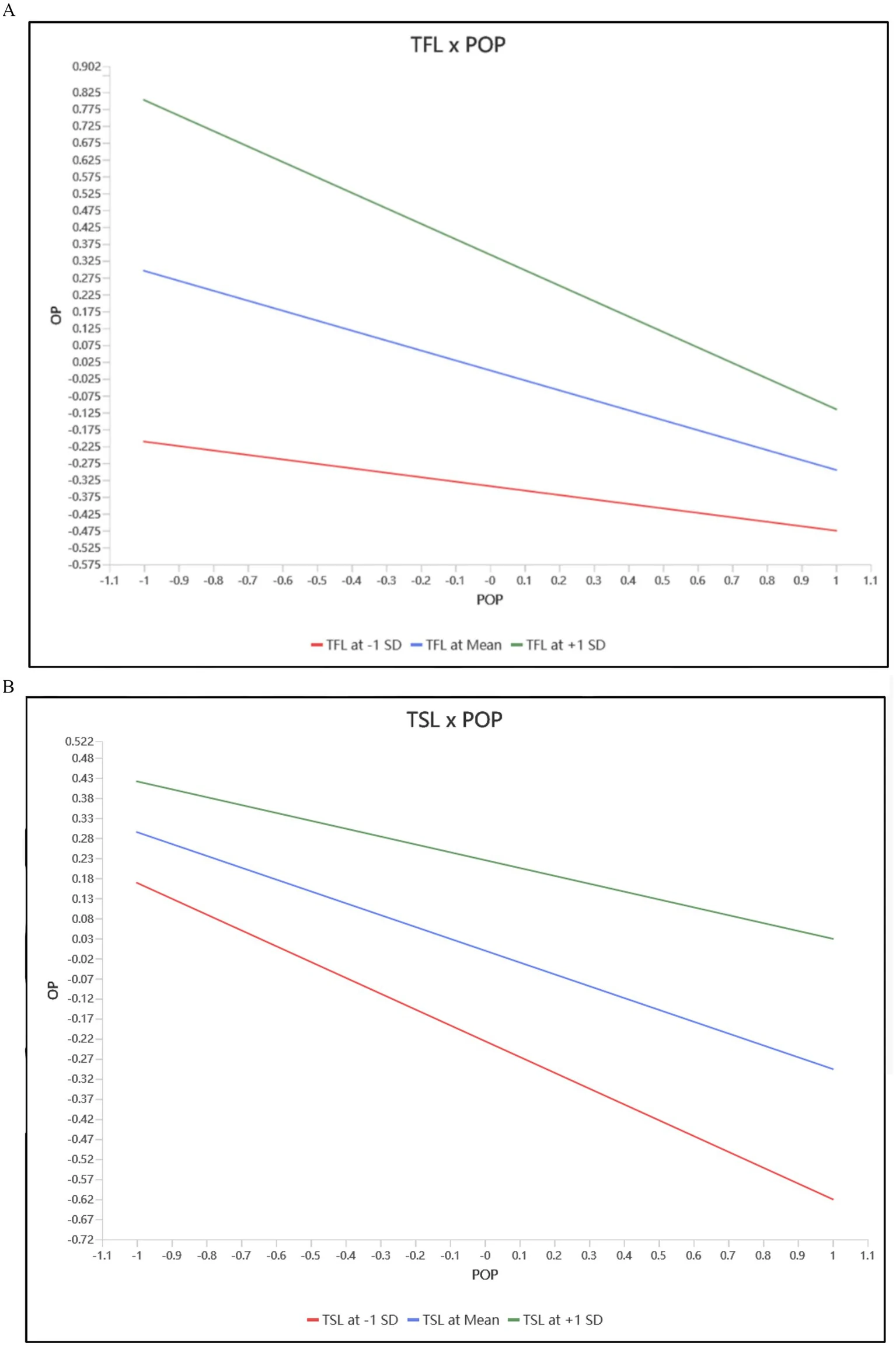
**(A)** Moderating effect of TFL between POP and OP. **(B)** Moderating effect of TSL between POP and OP.

## 5 Discussion

This study examined how transformational and transactional leadership influence the relationship between perceived organizational politics and organizational performance in governmental organizations. The findings confirm that perceived organizational politics had a negative and significant influence on organizational performance. This is consistent with previous literature, which has shown that high levels of POP are associated with negative outcomes, including diminished organizational performance, job satisfaction, employee outcomes, and organizational commitment (Asif et al., 2025; Cheong and Kim, 2017; Lee et al., 2021). The negative effects of POP on organizational performance are particularly evident in public organizations, where bureaucratic structures and political power dynamics often undermine the effectiveness, efficiency, and fairness of service delivery. Al-Okaily et al. (2024) found that organizational performance is significantly hindered by the negative consequences of POP within the public sector. Additionally, POP harms employees’ job satisfaction, increases burnout, fosters turnover intention, and reduces commitment, all of which contribute to reduced organizational performance (Vigoda-Gadot, 2007). Therefore, the findings confirm that perception of organizational politics negatively impacts organizational performance, specifically effectiveness, efficiency, and fairness within public organizations.

The moderating role of leadership styles has been confirmed between POP and OP. The results demonstrated that transformational leadership moderates the association between POP and OP. However, contrary to the original expectation, transformational leadership does not weaken the negative effect of POP. Instead, the interaction effect is negative, indicating that higher levels of transformational leadership strengthen the negative relationship between perceived organizational politics and organizational performance. This finding suggests that the effectiveness of transformational leadership may depend on the organizational context in which it is exercised. Although transformational leadership is generally associated with positive outcomes through vision, motivation, trust-building, and employee development (Bass and Riggio, 2006), these mechanisms may become less effective when employees perceive the organizational environment as politically driven.

One possible explanation is that transformational leadership emphasizes organizational values, collective goals, fairness, and shared vision. When employees perceive a discrepancy between these transformational values and actual organizational practices characterized by political influence, favoritism, or non-transparent decision-making, the inconsistency may become more salient. In such circumstances, transformational leadership may increase employees’ awareness of the gap between expected organizational principles and experienced political realities, thereby intensifying the negative consequences of POP. In other words, transformational leadership may raise employees’ expectations regarding fairness and integrity, making political behaviors perceived as particularly damaging when these expectations are violated. This interpretation extends previous research that has primarily emphasized the positive effects of transformational leadership and suggests that its effectiveness may be constrained under politically dysfunctional organizational conditions.

The finding also contributes to the leadership literature by highlighting the contextual boundary conditions of transformational leadership. Previous studies have shown that transformational leadership improves public-sector performance, promotes cooperation, and enhances employee outcomes (Asencio, 2016; Chau et al., 2022; Makatita et al., 2024). Kakhki et al. (2024) also found that TFL moderates the relationship between POP and knowledge management development, encouraging cooperative problem-solving. Additionally, TFL also moderates the negative effect of perception of organizational politics on physical, social, and environmental well-being (Sarpong et al., 2025). Transformational leaders focus on integrity, role modelling, innovation, coaching, and motivating employees, fostering a positive working environment that reduces the perception of organizational politics characterized by manipulation, personal interest, and favouritism (Dappa et al., 2019). Previous research also revealed that the negative impact of POP on organizational citizenship behaviors can be mitigated by TFL (De Clercq and Belausteguigoitia, 2017). These contextual factors bolster employees’ confidence in fulfilling their formal job responsibilities, notwithstanding the existence of dysfunctional political schemes (Bass and Riggio, 2006). However, the current findings indicate that transformational leadership should not automatically be interpreted as a universal remedy for politically perceived environments. Rather, its effectiveness depends on whether organizational structures and practices are consistent with the values promoted by transformational leaders.

Moreover, the formal comparison between the two interaction effects demonstrates that transformational and transactional leadership have significantly different moderating effects on the POP–OP relationship. Unlike the previous interpretation based only on individual significance levels, the coefficient difference test confirms that the two leadership styles operate differently under conditions of perceived organizational politics. The results indicate that transformational leadership produces a significantly stronger moderation effect than transactional leadership; however, this difference reflects a stronger negative amplification effect rather than a buffering effect. This finding advances previous leadership research by demonstrating that different leadership approaches do not simply vary in strength but may influence employees’ interpretations of politically charged organizational environments through distinct psychological mechanisms. Previous research suggests that TSL tends to focus on maintaining the status quo, which may limit its effectiveness in addressing the dynamic nature of organizational politics (Zafar, 2023). While transactional leadership can positively influence organizational performance in certain contexts (Nguyen et al., 2022), it appears less effective at mitigating the negative effects of POP compared to TFL. These findings are consistent with studies that have shown that POP did not moderate the relationships between transactional leadership and employees’ engagement (Dartey-Baah and Agbozo, 2021). This may be because transactional leadership, which emphasizes reward-based motivation, compliance with standard procedures, and performance monitoring, fails to address the root causes of political behaviors within organizations.

Additionally, the formal comparative analysis of the moderating effect of TFL and TSL revealed that transformational leadership plays a more significant moderating role in the relationship between POP and OP. The results indicate that transformational leadership significantly weakens the negative effect of POP on OP, whereas transactional leadership does not exhibit a statistically significant moderating effect. This finding supports prior studies that transformational leadership is generally more effective than transactional leadership in shaping favourable organizational outcomes and buffering adverse workplace conditions (Bass and Avolio, 2004; Ha and Moon, 2023). Transformational leaders create an environment that reduces political behaviors, fosters trust, and encourages collaboration, all of which are essential for improving organizational outcomes in public sector organizations. TSL is more transactional and control-oriented, so its buffering capacity is weaker.

From a practical perspective, the findings suggest that leadership development alone cannot fully resolve the negative consequences of organizational politics. Although transformational leadership is valuable for promoting trust, motivation, and engagement, managers should recognize that emphasizing organizational ideals without addressing underlying political problems may unintentionally heighten employees’ sensitivity to inconsistencies between organizational values and actual practices. Therefore, public organizations should prioritize institutional mechanisms that directly reduce perceptions of politics, including transparent decision-making procedures, equitable resource allocation, accountability systems, and merit-based promotion practices. Leadership development should complement, rather than substitute for, broader organizational reforms.

Finally, this study concludes that a substantial link exists among perceived organizational politics, organizational performance, and leadership styles in governmental organizations. The findings indicate that leadership styles play different and context dependent roles in shaping the relationship between POP and OP. The previous study suggest that TFL is an effective leadership styles that yield positive outcomes (Bao et al., 2024; Bass and Avolio, 2004; Serrat, 2021). The government service delivery can be made more efficient, effective, and responsive to the public through the implementation of TFL (Adanri and Thakkar, 2016). The findings challenge the assumption that transformational leadership always buffers negative workplace conditions and highlight the importance of considering organizational context when evaluating leadership effectiveness. In politically sensitive governmental environments, improving organizational fairness and transparency remains essential for enhancing performance outcomes.

### 5.1 Implications for theoretical and managerial

This study enhances the existing literature by offering valuable theoretical insights. First, the study presents a comprehensive model that incorporates the perceived organizational politics, organizational performance, and leadership styles. By examining these variables within a single model, the study advances understanding of how leadership styles shape the relationship between organizational politics and performance outcomes. Second, the findings enrich leadership theory by showing that leadership styles do not play equivalent roles in moderating the POP–OP relationship. Transformational leadership significantly weakens the negative effect of POP on organizational performance, whereas transactional leadership does not exert a significant moderating effect. This suggests that transformational leadership is a more effective contextual resource for reducing the harmful consequences of organizational politics and sustaining organizational effectiveness, efficiency, and fairness in public organizations.

This research also provides practical implications within the context of the public sector. First, the findings highlight the importance of addressing perceived organizational politics as a managerial issue, given its significant negative effect on organizational performance. Public organizations should therefore strengthen transparency, accountability, and fairness in organizational processes in order to reduce political perceptions and their detrimental consequences. Second, the results emphasize the practical value of transformational leadership in governmental organizations. Public managers should place greater emphasis on transformational leadership practices, such as articulating a clear vision, encouraging innovation, motivating employees, providing individualized support, and fostering collaboration (Ha and Moon, 2023). These leadership behaviors can help buffer the negative effects of organizational politics and improve public sector performance in terms of effectiveness, efficiency, and fairness. Third, public organizations do not rely only on a transactional control-oriented approach if their objective is to mitigate the negative influence of perceived organizational politics. Although transactional leadership may be useful for maintaining compliance, structure, and routine performance management, it does not significantly weaken the negative relationship between POP and organizational performance. Fourth, the study underscores the need for leadership development initiatives in governmental organizations. Policymakers and senior administrators should establish leadership training programs that strengthen transformational leadership capabilities, including motivation, inspiration, innovation, trust building, integrity, empowerment, and collaboration (Serrat, 2021). In addition, public organizations may benefit from incorporating leadership competency assessment into recruitment, promotion, and career development processes to ensure that managers are equipped to lead effectively in politically sensitive organizational environments.

### 5.2 Limitations and future research directions

Although this study significantly contributes to public management research, some limitations need to be noted. First, this study relied on single-source self-reported data from public managers. Transformational and transactional leadership were assessed using the MLQ leader self-report form, meaning that leadership scores reflected managers’ perceptions of their own behaviors rather than followers’ evaluations. In addition, perceived organizational politics and organizational performance were collected from the same respondents, which may increase the risk of common method bias. Although procedural and statistical measures were applied, this possibility cannot be entirely excluded. Future studies should use multi-source data, including subordinates, supervisors, and external stakeholders of leadership, and more objective measures of organizational performance. Second, the cross-sectional design limits the ability to draw conclusions about causality or to observe how leadership practices and perceptions of organizational politics evolve over time. Future research should undertake a longitudinal study to assess changes in leadership behavior and organizational outcomes, especially following leadership development interventions. Third, although the findings indicate that leadership styles influence the relationship between perceptions of organizational politics and organizational performance differently, this study examined only transformational and transactional leadership as moderating variables. This narrow focus limits a broader understanding of how other leadership approaches may operate in public organizations. Future research should explore additional emerging leadership styles, such as servant leadership, authentic leadership, and ethical leadership, to further understand their potential moderating roles.

## 6 Conclusion

This study provides empirical insights into the moderating effect of different leadership styles in the nexus between perceived organizational politics and organizational performance within the governmental organization. The results show that various leadership styles differ in their ability to moderate the relationship between POP and OP. Specifically, perceived organizational politics has a significant negative influence on organizational performance, highlighting the detrimental consequences of politically perceived organizational environments for public-sector effectiveness, efficiency, and fairness. The results further reveal that leadership styles do not influence the POP–OP relationship in the same manner. Transformational leadership was found to significantly moderate this relationship, however, contrary to the original expectation, it does not buffer the negative consequences of perceived organizational politics. Instead, the negative interaction effect indicates that higher levels of transformational leadership strengthen the adverse relationship between POP and OP. In contrast, transactional leadership did not show a significant moderating effect, indicating that reward-based exchange mechanisms and performance monitoring alone may be insufficient to address the negative consequences of perceived organizational politics. Furthermore, the comparative analysis confirms that the moderating effects of transformational and transactional leadership differ significantly. This finding extends previous research by demonstrating that leadership styles operate as context-dependent mechanisms rather than universally positive resources for overcoming organizational challenges. This study highlights the importance of leadership styles in shaping how organizational politics affects performance outcomes in the public sector. The study also offers practical and theoretical contributions. From a practical perspective, the findings suggest that government organizations should place greater emphasis on developing context-appropriate leadership styles, to reduce the harmful effects of organizational politics and strengthen organizational performance. From a theoretical perspective, the study extends the literature on perceived organizational politics and organizational performance by demonstrating the moderating role of leadership styles in a developing-country public sector context. Overall, this study contributes to understanding how leadership serves as a contextual resource for mitigating the adverse effects of organizational politics.

## Data Availability

The datasets presented in this study can be found in online repositories. The names of the repository/repositories and accession number(s) can be found in the article/Supplementary material.
