# Supplementary material for: Perceived organizational politics and organizational performance in governmental organizations: the moderating role of leadership styles
Source: Front Psychol. 2026 Sep 17;17:1838756. doi: 10.3389/fpsyg.2026.1838756 (PMC13627047; doi:10.3389/fpsyg.2026.1838756)
Supplement: Supplementary file 1 [file Supplementary_file_1.docx]

**Appendix**

**Perceived Organizational Politics and Organizational Performance in Governmental Organizations: The Moderating Role of Leadership Styles**

**Table of Contents**

Appendix A. Questionnaire	2

Appendix B. MLQ license	6

**Appendix A. Questionnaire**

**I.	Demographics Information**

1.1 What is your marital status?
- Single
- Married
- Divorce
  1.2 Please describe your age
  - Between 18 to 24
  - Between 25 to 34
  - Between 35 to 44
  - Between 45 to 54
  - Between 55 to 62
  1.3 Please describe your educational qualifications
  - Bachelor's Degree holder
  - Master's Degree holder
  - Doctoral Degree holder
  1.4 Please describe your working experience
- Between 11 to 15 years
- Between 16 to 20 years
- Between 21 to 25 years
- Between 26 to 30 years
- Between 31 and above
  1.5 Please describe your current duty station
- State and Regional Headquarters
- District Headquarters
- Township

**II	Perceived Organizational Politics**

Use the scale of: 5=Srongly Agree, 4=Agree, 3=Normal, 2=Disagree, 1=Strongly Disagree

| **No** | **Items** | **Question** | **1** | **2** | **3** | **4** | **5** |
| --- | --- | --- | --- | --- | --- | --- | --- |
| 1 | POP1 | Favoritism rather than merit determines who gets ahead around here |  |  |  |  |  |
| 2 | POP2 | Rewards come only to those who work hard in this organization |  |  |  |  |  |
| 3 | POP3 | There is a group of people in my department who always get things their way because no one wants to challenge them |  |  |  |  |  |
| 4 | POP4 | People in this organization attempt to build themselves up by tearing others down |  |  |  |  |  |
| 5 | POP5 | I have seen changes made in policies here that only serve the purposes of a few individuals, not the work unit or the organization |  |  |  |  |  |
| 6 | POP6 | People here usually don’t speak up for fear of retaliation by others |  |  |  |  |  |

**Source:** Vigoda, 2001

**III	Organizational Performance**

Use the scale of: 5=Srongly Agree, 4=Agree, 3=Normal, 2=Disagree, 1=Strongly Disagree

| **No** | **Items** | **Question** | 1 | 2 | 3 | 4 | 5 |
| --- | --- | --- | --- | --- | --- | --- | --- |
| 1 | IEFF | Last two years, the productivity of my work unit has improved. |  |  |  |  |  |
| 2 | IEFF | Overall, the quality of work my current coworkers perform in my immediate work group is high. |  |  |  |  |  |
| 3 | IEF | My organization has made good use of my knowledge and skills in looking for ways to become more efficient. |  |  |  |  |  |
| 4 | IEF | My organization is trying to reduce costs in managing the organization and performing work. |  |  |  |  |  |
| 5 | IF | My organization provides fair and equitable treatment for employees and applicants in all aspects of personnel management without regard to their political affiliation, sex, hometown, marital status, age, or handicapping condition. |  |  |  |  |  |
| 6 | IF | Generally, all are treated with respect in my organization, with no regard to status or grade. |  |  |  |  |  |
| 7 | EEFF | The work performed by my work unit provides the public with a worthwhile return on their tax money. |  |  |  |  |  |
| 8 | EEFF | The occurrence of goal attainment is very high in my organization |  |  |  |  |  |
| 9 | EEF | My organization has conducted business relations with outside customers very promptly. |  |  |  |  |  |
| 10 | EEF | It is rare to make big mistakes in my organization when conducting work |  |  |  |  |  |
| 11 | EF | My organization provides fair and equitable services to the public without considering their backgrounds. |  |  |  |  |  |
| 12 | EF | The customer satisfaction with my organization is very high |  |  |  |  |  |

**Source:** Kim, 2005

**IV	Leadership Styles (MLQ-5X)**

Use the scale of: 0=Not at all, 1=Once in a while, 2=Sometimes, 3=Fairly often, 4=Frequently, if not always

| **No** | **Items** | **Question** | **0** | **1** | **2** | **3** | **4** |
| --- | --- | --- | --- | --- | --- | --- | --- |
| 1 | TFL | Idealized influence (Attribute) |  |  |  |  |  |
| 2 | TFL | Idealized influence (Attribute) |  |  |  |  |  |
| 3 | TFL | Idealized influence (Attribute) |  |  |  |  |  |
| 4 | TFL | Idealized influence (Attribute) |  |  |  |  |  |
| 5 | TFL | Idealized influence (Behavior) |  |  |  |  |  |
| 6 | TFL | Idealized influence (Behavior) |  |  |  |  |  |
| 7 | TFL | Idealized influence (Behavior) |  |  |  |  |  |
| 8 | TFL | Idealized influence (Behavior) |  |  |  |  |  |
| 9 | TFL | Inspirational Motivation |  |  |  |  |  |
| 10 | TFL | Inspirational Motivation |  |  |  |  |  |
| 11 | TFL | Inspirational Motivation |  |  |  |  |  |
| 12 | TFL | Inspirational Motivation |  |  |  |  |  |
| 13 | TFL | Intellectual Stimulation |  |  |  |  |  |
| 14 | TFL | Intellectual Stimulation |  |  |  |  |  |
| 15 | TFL | Intellectual Stimulation |  |  |  |  |  |
| 16 | TFL | Intellectual Stimulation |  |  |  |  |  |
| 17 | TFL | Individual Consideration |  |  |  |  |  |
| 18 | TFL | Individual Consideration |  |  |  |  |  |
| 19 | TFL | Individual Consideration |  |  |  |  |  |
| 20 | TFL | Individual Consideration |  |  |  |  |  |
| 21 | TSL | Management by Exception (Active) |  |  |  |  |  |
| 22 | TSL | Management by Exception (Active) |  |  |  |  |  |
| 23 | TSL | Management by Exception (Active) |  |  |  |  |  |
| 24 | TSL | Management by Exception (Active) |  |  |  |  |  |
| 25 | TSL | Contingent Reward |  |  |  |  |  |
| 26 | TSL | Contingent Reward |  |  |  |  |  |
| 27 | TSL | Contingent Reward |  |  |  |  |  |
| 28 | TSL | Contingent Reward |  |  |  |  |  |

**Source:** Mind Garden, n.d.; Bass & Avolio, 2004; Serrat, 2021

**Note:** This is a sample question. The study utilized MLQ5X standardized questions. Due to licensing restrictions, the full set of MLQ5X questions is not included in the appendix. However, they can be obtained from the source or by contacting the authors.

**Appendix B. MLQ License**
